# Oxidative and Epigenetic Changes and Gut Permeability Response in Early-Treated Chickens with Antibiotic or Probiotic

**DOI:** 10.3390/ani10122204

**Published:** 2020-11-25

**Authors:** Katarzyna Ognik, Paweł Konieczka, Anna Stępniowska, Jan Jankowski

**Affiliations:** 1 Department of Biochemistry and Toxicology, Faculty of Animal Science and Bioeconomy, University of Life Sciences, Akademicka 13, 20-950 Lublin, Poland; anna.stepniowska@up.lublin.pl; 2 Department of Poultry Science, Faculty of Animal Bioengineering, University of Warmia and Mazury in Olsztyn, Oczapowskiego 5, 10-719 Olsztyn, Poland; pawel.konieczka@uwm.edu.pl (P.K.); janj@uwm.edu.pl (J.J.)

**Keywords:** chicken, antibiotic, probiotic, intestinal damage

## Abstract

**Simple Summary:**

‘Prophylactic’ use of antibiotics is quite common, but is increasingly controversial due to their potentially negative effects on birds. The excessive use of antibiotics may lead to the spread of drug-resistant infections in both birds and humans. The potential threat arising from intensification of the oxidative stress reaction and immune system impairment in birds due to the use of antibiotics is still not sufficiently understood. The aim of this study was to compare the effect of the use of enrofloxacin and a probiotic containing *Enterococcus faecium* and *Bacillus amyloliquefaciens* strains in the first week of life of chickens on intestinal integrity. Our research indicated that the use of both enrofloxacin similar to a probiotic containing *Enterococcus faecium* and *Bacillus amyloliquefaciens* strains in chickens’ first week of life improved intestinal integrity and reduced inflammation in the small intestine.

**Abstract:**

The aim of this study was to compare the effect of the use of enrofloxacin and a probiotic containing *Enterococcus faecium* and *Bacillus amyloliquefaciens* strains in the first week of life of chickens on oxidative and epigenetic changes in molecules and intestinal integrity. The three treatments were as follows: the control group received no additive in the drinking water (GC); the second group (GP) received a probiotic preparation in the drinking water during the first five days of life, providing *E. faecium* strain 4a1713 at 1.0 × 10^7^ CFU/L water and *B. amyloliquefaciens* 4b1822 at 1.0 × 10^7^ CFU/L water, the third group (GA) received an antibiotic (enrofloxacin 0.5 mL/L water) in the drinking water during the first five days of life. The use of both enrofloxacin and a probiotic containing *E. faecium* and *B. amyloliquefaciens* strains in chickens’ first week of life improved intestinal integrity and reduced inflammation and oxidative and epigenetic changes in the small intestine. This effect was evident both at 6 days of age and at the end of the rearing period.

## 1. Introduction

In the early life of farmed birds, disturbances in gastrointestinal function are common. Clinical enteropathies are estimated to constitute up to 50% of pathologies in broilers and fattening turkeys. Their most common causes are bacterial and viral infections. To prevent enteropathy, various feed additives are used in the first few days of life [1,2,3]. These are mainly probiotics facilitating the colonization of the digestive tract by beneficial bacteria [4,5]. ‘Prophylactic’ use of antibiotics is also quite common, but is increasingly controversial due to their potentially negative effects on birds (affecting immune and antioxidant function or causing epigenetic changes). The potential threat arising from intensification of the oxidative stress reaction and immune system impairment in birds due to the use of antibiotics is still not sufficiently understood.

One of the most commonly used antibiotics in metaphylaxis is enrofloxacin, which has a broad spectrum of activity against bacteria, including *Escherichia coli*, *Pasteurella* spp., *Salmonella* spp. and *Mycoplasma* spp., in poultry [6]. The use of enrofloxacin in chickens’ first days of life has been shown to stimulate the immune response, especially through modification of the proportions of lymphocyte subsets in lymphoid organs, an effect which persists until the end of the rearing period [7]. Similarly, research by Hassanin et al. [8], Khalifeh et al. [6] and Tokarzewski [9] demonstrates that the use of enrofloxacin modifies the immune response of birds by weakening humoral immunity consisting in antibody production and stimulating a cellular response involving T cells as the main effector cells. Data presented by Laxminarayan and Heymann [10] indicate that antimicrobial resistance in bacteria is a consequence of the use of antibiotics. The excessive use of antibiotics may lead to the spread of drug-resistant infections in both birds and humans [11]. Many authors therefore suggest using probiotics to reduce the need for antibiotics in poultry farming [11,12,13,14]. Most probiotics are bacteria colonizing the gastrointestinal tract of animals and act as bacterial community stabilizers or antimicrobials against undesirable bacterial species [15,16,17]. Previous research shows that the use of probiotics in poultry nutrition improves gut health by modulating the intestinal microbiota and nutrient absorption and also strengthens the immune and antioxidant system [14,18,19,20]. Probiotics are believed to have a beneficial effect on the gut microbiota by limiting the colonization of the gut by harmful microorganisms while promoting the growth of beneficial microorganisms [21,22]. In contrast, antibiotics reduce intestinal colonization by both harmful and saprophytic microorganisms [5]. There are reports indicating that intestinal colonization by microorganisms affects protein and DNA oxidation and epigenetic changes in DNA not only in the gut, but in other tissues as well [23]. Negative oxidative and epigenetic changes may result in the induction of intestinal inflammation [24,25,26,27,28], which in turn can directly disturb the integrity of the intestinal barrier [28]. The aim of this study was to compare the effect of the use of enrofloxacin and a probiotic containing *Enterococcus faecium* and *Bacillus amyloliquefaciens* strains in the first week of life of chickens on oxidative and epigenetic changes in molecules and intestinal integrity.

## 2. Material and Methods

### 2.1. Chicken Experiment

The experiment (preliminary research) was carried out on 90 one-day-old male broilers of the Ross 308 strain with an initial body weight of 42.8 g ± 0.90, purchased from a local commercial hatchery. The birds were randomly assigned to three dietary treatments with 30 birds in each and placed in pens on litter. The housing conditions were in accordance with standard management practices in a commercial chicken house. The birds were fed commercial starter and grower diets (Table 1) formulated to meet or exceed their nutritional requirements in accordance with their age [29]. The three treatments were as follows: the control group received no additive in the drinking water (GC); the second group (GP) received a probiotic preparation in the drinking water during the first five days of life, providing *E. faecium* strain 4a1713 at 1.0 × 10^7^ CFU/L water and *B. amyloliquefaciens* 4b1822 at 1.0 × 10^7^ CFU/L water, as stated by the manufacturer (BioPoint, Poland); the third group (GA) received an antibiotic (enrofloxacin 0.5 mL/L water) in the drinking water during the first five days of life. The drinking water in each group was changed daily, and a fresh preparation of either the probiotic supplement or the antibiotic was applied during the five-day period. The birds were reared until the age of 35 days, and their body weight and feed intake were measured. The protocol for the study was in compliance with the guidelines laid down by the European Union and the Polish Law on Animal Protection.

### 2.2. Sampling Procedures

At 6 and 35 days of age, chickens were weighed individually, and a total of 10 birds from each dietary treatment were randomly selected and sacrificed by cervical dislocation, preceded by electric stunning. Blood and intestinal samples were taken from 10 birds from each group. Blood for analysis was collected from the wing vein into test tubes with an anticoagulant (heparin). Next the blood samples were centrifuged at 3000× *g* for 10 min and the plasma was collected for further analysis. 8-hydroxydeoxyguanosine (8-OHdG), endonuclease 1 (APE-1), and oxoguanine glycosylase (OGG1 protein) were determined in the blood and intestinal wall using OxiSelect diagnostic kits (Cell Biolabs, Inc., San Diego, CA, USA). DNA was isolated from the blood and intestinal wall using kits manufactured by QIAGEN. The level of epigenetic changes in the blood and intestinal wall of the chickens was determined by analysing global DNA methylation (methylome) using diagnostic kits manufactured by Sigma Aldrich. OxiSelect diagnostic kits (Cell Biolabs, Inc., San Diego, CA, USA) were used to determine protein carbonyl (PC) derivatives as an indicator of the oxidation of amino acid residues. Caspase 3, Caspase 8, diamine oxidase (DAO) and lactic acid (LA) content were determined in the blood and intestinal wall using an ELISA kit (Cell Biolabs, Inc. San Diego, CA, USA). The plasma level of the CRP protein was determined in an ELISA reader using assays from Elabscience Biotechnology Co., Ltd. (Houston, TX, USA).

### 2.3. Ethical Statement

All procedures involved handling the birds were performed by qualified veterinarians. No action involving pain or suffering was practiced, and all of the analyses were performed on samples collected post-mortem. The protocol for this study and the number of chickens used in this study were consistent with the regulations of the Local Committee for Experimentation on Animals (Olsztyn, Poland) and were performed in accordance with the principles of the European Union Directive 2010/63/EU for animal experiments and Polish Law on Animal Protection. Accordingly to directive no. 2010/63/EU the approval of the Ethics Committee was not required. The directive states that the requirements for the protection of animals used for experimental purposes. There it is described that these rules do not apply to agricultural activities and animal husbandry. The experiment was conducted in commercial conditions, so farmers were responsible for rearing. In addition, there is resolution 13/2016 of the National Ethics Committee for Animal Experiments of 17 June 2016, where: Collecting material from animals in breeding for genotyping and marking these animals are not procedures within the meaning of the Act on the protection of animals used for scientific or educational purposes and you do not need to obtain the consent of the Local Ethics Committee.

### 2.4. Statistical Analysis

Data are presented as the mean ± standard error of the mean (SEM) (*n* = 10 for each group). Differences between groups were determined by one-way ANOVA using Tukey’s HSD test. The significance level was set at *p* < 0.05. All calculations were performed using the GLM procedure of STATISTICA software version 12. Data for feed consumption were calculated using each pen as a replicate.

## 3. Results

The mean body weight of the birds at 6 days of age did not differ significantly (*p* = 0.571) and was 0.152 kg ± 0.009, 0.156 kg ± 0.009 and 0.157 kg ± 0.013 for treatments C, GP and GA, respectively. At 35 days of age, the final average body weight for groups GC, GP and GA was 2.37 kg ± 0.109, 2.52 kg ± 0.211 and 2.33 kg ± 0.281, respectively, and was significantly higher in birds receiving the probiotic in their drinking water than in the other groups (*p* = 0.049). The mean feed intake for the period from day 1 to day 6 was 0.169 kg, 0.163 kg and 0.162 kg for groups GC, GP and GA, respectively.

Compared to chickens from the GC treatment, both the GP and GA treatments reduced the plasma level of CRP in chickens at 35 d of age (Table 2). At both 6 and 35 d of age, DAO activity in the small intestinal wall was lower in the GP and GA treatments (*p* < 0.001, both) than in the chickens from the GC treatment. Compared to the GC treatment, 35-day-old chickens from the GP treatment also showed decreased plasma activity of this enzyme (*p* = 0.016). Chickens from the GP treatment had lower LA levels in the wall of the small intestine at both 6 and 35 days of age (*p* = 0.011; *p* = 0.035, respectively) than GC and GA chickens. A decreased LA level in the plasma of chickens from the GP treatment was also noted at 35 days of age (*p* = 0.004; Table 2, Table 3 and Table 4). In the small intestinal wall of chickens from the GP and GA treatments at 6 d of age, higher PC levels (*p* = 0.042) were noted than in the GC treatment. Chickens from the GP treatment had an increased level of 8-OHdG in the small intestinal wall compared to chickens from the GC and GA treatments (*p* = 0.004), but only at 35 days of age (Table 3 and Table 4). Compared to the GC treatment, 6-day-old chickens from the GP and GA treatments had a reduced percentage of methylated DNA in the small intestinal wall (*p* < 0.001). At 35 days of age, the reduced percentage of methylated DNA persisted in both the small intestinal wall (*p* < 0.001) and the blood plasma (*p* = 0.001) only in case of the GA treatment (Table 2, Table 3 and Table 4). In the small intestinal wall of 35-day-old chickens from the GP treatment (*p* < 0.001), the percentage of methylated DNA was increased relative to the GC treatment. In 6-day-old chickens from the GP treatment, levels of OGG1 and APEX-1 in the small intestinal wall were lower (*p* < 0.001, both) than in the chickens from the GC treatment. In 35-day-old chickens, decreased levels of OGG1 and APEX-1 in the wall of the small intestine were noted in both the GP and GA treatments (*p* < 0.001, both; Table 3 and Table 4). Compared to the GC treatment, the GA treatment increased the level of Casp 3 in the small intestinal wall (*p* = 0.024) at 6 days of age. In the small intestinal wall of 35-day-old chickens from the GA treatment, decreased levels of both Casp 3 (*p* = 0.003) and Casp 8 (*p* < 0.001) were noted relative to the GC treatment (Table 3 and Table 4). A decreased level of Casp 8 (*p* = 0.038) was noted in the plasma of GA chickens compared to chickens from the GP treatment.

## 4. Discussion

Available literature data indicate that antimicrobial preparations and other modulators of the gut microbiome, such as probiotics, yeast products, organic acids, essential oils, and enzymes, do not improve rearing performance when animals are kept in optimal environmental conditions [31,32]. In our research, the addition of enrofloxacin to the drinking water of chickens in the first week of rearing did not affect growth performance, as the final body weight was comparable to that noted in the control group. Similarly, da Costa et al. [33] administered enrofloxacin to chickens in their first three days of life and found no effect on growth performance. The fact that this antibiotic has not significantly affected rearing results can be explained by the lack of harmful intestinal bacteria in one-day-old chickens [31]. Krauze et al. [14] administered a probiotic containing *Bacillus subtilis* or *E. faecium* to chickens throughout the rearing period and noted no effect on production results. In contrast, in our research the use of a probiotic supplement containing *E. faecium* and *B. amyloliquefaciens* strains in the drinking water of chickens during the first week of rearing improved growth performance. Similarly, Ognik et al. [20] observed improved rearing results in chickens receiving a probiotic containing *E. faecium* throughout the rearing period.

In large-scale poultry rearing, especially in the first few days, infectious agents have a significant impact on the functional state of the intestines and immune system, although environmental conditions and stress also play a role. Disturbances in the function of the intestinal barrier may increase its permeability, resulting in the induction of oxidative and pro-inflammatory reactions [34]. When intestinal epithelial cells are damaged, adhesion of leukocytes and damage to the intestinal endotheliocytes will increase concentrations of diamine oxidase and D-lactate [35,36]. Diamine oxidase is an enzyme found in the cells of the intestinal mucosa. It is released into the bloodstream during enterocyte damage, and thus increased DAO levels indicate increased intestinal permeability [37]. Wu et al. [38] and Li et al. [39] reported an elevated serum DAO level in LPS-challenged broiler chickens. In our study, the use of the antibiotic enrofloxacin or a probiotic containing *E. faecium* and *B. amyloliquefaciens* strains in the first week of rearing reduced DAO activity in the small intestinal wall of the chickens, and this effect persisted until the end of the rearing period. According to Fernandes et al. [40], probiotics increase gut integrity in chickens by favourably modulating the gut microbiota and reducing inflammation. In a study by Zhang et al. [4], the use of a probiotic containing *Clostridium butyricum* or the antibiotic colistin in the diet of *E. coli*-challenged chickens resulted in a decrease in DAO activity. The authors of that study found that the probiotic containing *C. butyricum* was more effective than colistin in protecting the gut against disturbance of intestinal barrier function. Our research also indicates that the probiotic had a more beneficial effect than the antibiotic on the integrity of the intestinal barrier. Decreased LA levels were noted in the small intestinal wall and the blood plasma of chickens receiving the probiotic containing *E. faecium* and *B. amyloliquefaciens*, which was not observed in the case of enrofloxacin. Research cited by Taylor [41] indicates that the composition of the diet can modify the level of LA in the gut of laying hens, and the beneficial effect of probiotics reduces this parameter.

Carbonyl derivatives are most often formed by the oxidation of amino acid residues, especially proline, arginine, lysine and threonine. Accumulated carbonyl derivatives of damaged proteins are markers of the degree of intensification of protein oxidation [42]. In our research, the use of a probiotic containing *E. faecium* and *B. amyloliquefaciens* or enrofloxacin in the first week of chickens’ life resulted in an increase in the level of PC in the small intestinal wall immediately following the use of the experimental additives. However, on the birds’ last day of life, elevated PC levels were not noted in either the small intestinal wall or the blood plasma of chickens receiving either the probiotic or the antibiotic. Literature data show that probiotics exhibit antioxidant activity [14,19,43]. Xue et al. [43], in a study on laying hens receiving a probiotic containing *B. subtilis* strains, found that this supplement had no effect on the PC level in the muscles.

In the present study, the use of a probiotic supplement containing *E. faecium* and *B. amyloliquefaciens* strains or enrofloxacin in the first week of rearing of chickens did not result in the induction of DNA oxidation, as evidenced by the balanced level of 8-OHdG in the small intestinal wall of the 6-day-old chickens. Moreover, both the probiotic supplement and the antibiotic reduced the percentage of methylated DNA in the small intestine of 6-day-old chickens, but this effect persisted until the end of the rearing period only in the chickens that had received the antibiotic. However, it is difficult to explain the increased level of 8-OHdG and percentage of DNA methylation in the small intestinal wall of 35-day-old chickens receiving the probiotic, especially as there was no increase in these parameters in the plasma of birds from this group. 8-OHdG is a biomarker of DNA oxidative damage whose level may be increased by hydroxylation of guanosine DNA residues [44]. DNA methylation is an epigenetic mechanism that inhibits DNA transcription through the addition of methyl residues to cysteine. DNA methylation can be modulated by environmental factors such as diet, stress, climate, and the gut microbiota [23,45,46,47]. Literature data show that the level of DNA methylation may be influenced by the short-chain fatty acid butyrate, which is a product of prebiotic fermentation [48,49]. According to Dunisławska et al. [23], early stimulation of the gut microbiota of chickens has a strong effect leading to permanent changes in the DNA methylation profile. There is research showing that DNA damage can be reduced by the use of antioxidants. Studies on rats have shown that the administration of probiotic bacteria has genoprotective potential [50]. The authors of that study noted a decrease in the level of 8-OHdG as a result of the use of *Lactobacillus* in the diet.

The OGG1 protein is the main DNA glycosylase for the repair of 8-OHdG lesions in DNA [51]. Base excision repair (BER) in DNA involves the recognition and removal of the defective base by DNA glycosylases (OGG1) to create an apurinic/apyrimidinic (AP) site. This site is then recognized by apurinic/apyrimidinic endonuclease (APEX-1), which hydrolyses the phosphodiester backbone to the AP site to form a 3′-OH group, which can then be recognized by DNA polymerase [52]. In our research, the use of a probiotic supplement containing *E. faecium* and *B. amyloliquefaciens* in the first week of chicken rearing reduced the level of DNA repair enzymes (OGG1 and APEX-1) in the small intestinal wall, an effect that persisted until the end of rearing. Interestingly, in the case of the antibiotic enrofloxacin, the levels of OGG1 and APEX-1 in the small intestinal wall were initially (at 6 d of age) similar to those recorded in the control group, but had decreased by the end of the rearing period (day 35), by about 50% compared to the control group. This suggests that the use of neither the probiotic nor the antibiotic in the first week of rearing caused DNA damage. The use of the antibiotic enrofloxacin in the first week of rearing raised the level of Casp 3 in the small intestinal wall of 6-day-old chickens. However, on the last day of rearing of chickens receiving the antibiotic, a decrease was noted in levels of Casp 3 in the small intestinal wall and of Casp 8 in the small intestinal wall and the blood plasma. According to Fischer et al. [53], antibiotics may have pro-apoptotic effects. Apoptosis, or programmed cell death, is induced by the caspase cascade, in particular by Casp-3, which catalyses the specific cleavage of many key cellular proteins and nuclear DNA [54]. Moreover, Casp-3 inhibits the release of pro-inflammatory mediators by cleaving off transcription factor NF-B [53]. This mechanism explains the anti-inflammatory effect of antibiotics. C-reactive protein (CRP) is a marker of systemic inflammation whose level increases during a prolonged inflammatory response [55]. In our study, the use of enrofloxacin or a probiotic containing *E. faecium* and *B. amyloliquefaciens* in the first week of rearing reduced inflammation in chickens, which was expressed by a reduced CRP level in the blood on the last day of rearing. Pomorska-Mól et al. [56] reported that enrofloxacin lowered the level of pro-inflammatory cytokines and acute phase proteins, thereby reducing the inflammatory response. The anti-inflammatory and immunomodulatory properties of enrofloxacin have also been confirmed by Chrząstek and Wieliczko [7] in a study on chickens. There are reports indicating that some strains of probiotic bacteria also reduce inflammation of the intestinal mucosa by modulating the level of pro-inflammatory cytokines and other inflammatory mediators [57,58]. In studies by Krauze et al. [14] and Abramowicz et al. [19], the use of a probiotic containing *Bacillus subtilis* in the diet of chickens reduced the level of pro-inflammatory interleukin 6.

## 5. Conclusions

The use of both enrofloxacin and a probiotic containing *E. faecium* and *B. amyloliquefaciens* strains in chickens’ first week of life improved intestinal integrity and reduced inflammation and oxidative and epigenetic changes in the small intestine. This effect was evident both at 6 days of age and at the end of the rearing period.

## Figures and Tables

**Table 1:** Composition of starter and grower diets, g/100g.

Ingredients	Starter (1–14 Days)	Grower (15–35 Days)
Wheat	64.071	64.586
Soybean meal	28.772	21.085
Rapeseed meal	-	50.0
Soybean oil	3.232	5.868
Limestone	1.349	1.277
L-Lysine	0.403	0.318
DL-Methionine	0.301	0.227
L-Threonine	0.077	0.081
MCP	0.956	0.742
NaCl	0.339	0.316
Vitamin and mineral premix ^1^	0.50	0.50
Calculated analysis ^2^		
AMEn MJ/kg	12.35	12.98
Crude protein	24.97	29.13
Lysine	1.35	1.16
Methionine	0.604	0.517
Met. + Cys.	1.00	0.900
Threonine	0.830	0.770
Ca	0.900	0.850
P	0.637	0.590
P available	0.400	0.350

^1^ Provides per kg feed: IU: vit. A. 10,000, vit. D_3_ 4500; mg: vit. E 80, vit. B_1_ 1.5, vit. B_2_ 5, biotin 0.12, vit. B_6_ 2.5, vit. B_12_ 0.02, vit. K_3_ 3, nicotinic acid 50, folic acid 1.1, pantothenic acid 14, choline 200, betaine 160, Mn 120, Zn 100, Se 0.35, Cu 20, Fe 40, J 3, Ca 0.6 g., phytase 1000 FTU. ^2^ Calculated according to Polish Feedstuff Analysis Tables [30].

**Table 2:** Oxidative and epigenetic DNA damage and activity of repair enzymes in the blood of chickens (35 day of life).

Item	Casp 3 ng/mL	Casp 8 ng/mL	8-OHdG ng/mL	OGG1 ng/mL	APEX-1 ng/L	DAO U/L	LA mmol/L	PC nmol/mg Protein	% DNA Methylation	CRP mg/dL
Treatment ^1^										
GC	0.127	35.97 ^ab^	0.742	18.87	216.0	9.486 ^a^	0.328 ^a^	3.023	72.38 ^a^	1.210 ^a^
	±0.057	±3.89	±0.109	±6.55	±75.1	±2.400	±0.059	±0.745	±8.34	±0.100
GP	0.139	39.07 ^a^	0.746	25.93	231.8	8.077 ^ab^	0.220 ^b^	2.524	71.27 ^a^	1.094 ^b^
	±0.066	±4.48	±0.197	±9.39	±94.0	±1.265	±0.048	±0.303	±6.25	±0.071
GA	0.145	32.75 ^b^	0.728	22.27	267.0	7.186 ^b^	0.345 ^a^	3.119	58.79 ^b^	1.067 ^b^
	±0.097	±6.78	±0.221	±4.27	±82.2	±0.989	±0.120	±0.523	±9.04	±0.044
SEM	0.013	1.034	0.032	1.354	15.34	0.342	0.018	0.113	1.810	0.018
*p*-value	0.864	0.038	0.973	0.101	0.394	0.016	0.004	0.063	0.001	<0.001

^a,b^ Means within the same column differ significantly (*p* ≤ 0.05) according to Tukey’s HSD test ^1^ Treatment: GC received no additive in the drinking water; GP received a probiotic preparation, providing *Enterococcus faecium* strain 4a1713 at 1.0 × 107 CFU/L water and *Bacillus amyloliquefaciens* 4b1822 at 1.0 × 107 CFU/L water; GA received enrofloxacin 0.5 mL/L water. Casp 3—Caspase 3, Casp 8—Caspase 8, 8-OHdG-*8-Hydroxydeoxyguanosine,* OGG1—protein oxoguanine glycosylase, APE-1—endonuclease 1, DAO—diamine oxidase, LA—lactic acid, PC—protein carbonyl, CRP—C reactive protein.

**Table 3:** Oxidative and epigenetic DNA damage and activity of repair enzymes in intestinal wall of chickens (6 day of life).

Item	Casp 3 ng/g	Casp 8 ng/g	8-OHdG ng/g	OGG1 ng/g	APEX-1 ng/g	DAO U/g	LA mmol/g	PC nmol/mg Protein	% DNA Methylation
Treatment ^1^									
GC	30.28 ^b^ ± 9.74	48.57 ± 6.45	39.36 ± 14.31	43.27 ^a^ ± 9.70	2791.3 ^a^ ± 548.5	0.165 ^a^ ± 0.060	0.015 ^a^ ± 0.006	40.08 ^b^ ± 6.77	41.17 ^a^ ± 4.29
GP	32.13 ^ab^ ± 9.09	49.57 ± 9.91	49.10 ± 18.20	24.59 ^b^ ± 9.76	1545.4 ^b^ ± 537.7	0.069 ^b^ ± 0.031	0.009 ^b^ ± 0.003	47.92 ^a^ ± 7.70	34.08 ^b^ ± ±4.21
GA	41.97 ^a^ ± 9.89	54.88 ± 12.65	43.18 ± 15.26	33.38 ^ab^ ± 9.74	2162.1 ^ab^ ± 613.0	0.109 ^b^ ± 0.041	0.011 ^ab^ ± 0.003	48.35 ^a^ ± 8.72	30.55 ^b^ ± 5.34
SEM	1.939	1.834	2.917	2.225	137.5	0.011	0.001	1.540	1.158
*p*-value	0.024	0.331	0.403	<0.001	<0.001	<0.001	0.011	0.042	<0.001

^a,b^ Means within the same column differ significantly (*p* ≤ 0.05) according to Tukey’s HSD test ^1^ Treatment: GC received no additive in the drinking water; GP received a probiotic preparation, providing *Enterococcus faecium* strain 4a1713 at 1.0 × 107 CFU/L water and *Bacillus amyloliquefaciens* 4b1822 at 1.0 × 107 CFU/L water; GA received enrofloxacin 0.5 mL/L water. Casp 3—Caspase 3, Casp 8—Caspase 8, 8-OHdG—*8-Hydroxydeoxyguanosine*, OGG1—protein oxoguanine glycosylase, APE-1—endonuclease 1, DAO—diamine oxidase, LA—lactic acid, PC—protein carbonyl.

**Table 4:** Oxidative and epigenetic DNA damage and activity of repair enzymes in intestinal wall of chickens (35 day of life).

Item	Casp 3 ng/g	Casp 8 ng/g	8-OHdG ng/g	OGG1 ng/g	APEX-1 ng/g	DAO U/g	LA mmol/g	PC nmol/mg Protein	% DNA Methylation
Treatment ^1^									
GC	23.58 ^a^ ± 2.66	27.12 ^a^ ± 5.76	28.50 ^b^ ± 7.65	48.81 ^a^ ± 7.92	2940.3 ^a^ ± 412.6	0.159 ^a^ ± 0.015	0.012 ^a^ ± 0.004	82.70 ± 14.18	36.97 ^b^ ± 5.06
GP	19.33 ^ab^ ± 6.76	26.40 ^a^ ± 3.72	39.43 ^a^ ± 5.55	33.61 ^b^ ± 8.41	2094.6 ^b^ ± 430.4	0.075 ^b^ ± 0.016	0.007 ^b^ ± 0.004	83.47 ± 15.65	44.85 ^a^ ± 8.95
GA	15.85 ^b^ ± 3.33	9.43 ^b^ ± 4.47	34.59 ^ab^ ± 6.24	24.81 ^c^ ± 7.41	1422.2 ^c^ ± 594.8	0.084 ^b^ ± 0.038	0.010 ^ab^ ± 0.005	79.39 ± 17.82	28.49 ^c^ ± 4.70
SEM	1.003	1.731	1.420	2.311	143.7	0.008	0.001	2.830	1.692
*p*-value	0.003	<0.001	0.004	<0.001	<0.001	<0.001	0.036	0.832	<0.001

^a,b,c^ Means within the same column differ significantly (*p* ≤ 0.05) according to Tukey’s HSD test ^1^ Treatment: GC received no additive in the drinking water; GP received a probiotic preparation, providing *Enterococcus faecium* strain 4a1713 at 1.0 × 107 CFU/L water and *Bacillus amyloliquefaciens* 4b1822 at 1.0 × 107 CFU/L water; GA received enrofloxacin 0.5 mL/L water. Casp 3—Caspase 3, Casp 8—Caspase 8, 8-OHdG—*8-Hydroxydeoxyguanosine*, OGG1—protein oxoguanine glycosylase, APE-1—endonuclease 1, DAO—diamine oxidase, LA—lactic acid, PC—protein carbonyl.
